# CircUCP2 promotes the tumor progression of non-small cell lung cancer through the miR-149/UCP2 pathway

**DOI:** 10.32604/or.2023.030611

**Published:** 2023-09-15

**Authors:** WEI DU, FANG YIN, YATING ZHONG, MINJIE LUO, ZHEN WANG, PENG LIN, QING LIU, HAN YANG

**Affiliations:** 1 Department of Pathology, The First People’s Hospital of Changde City, Changde, 415000, China; 2 State Key Laboratory of Oncology in South China, Sun Yat-sen University Cancer Center, Guangzhou, 510000, China

**Keywords:** CircUCP2, miR-149, UCP2, CeRNAs, NSCLC

## Abstract

Non-small cell lung cancer (NSCLC) is a highly lethal cancer, and better treatments are urgently needed. Many studies have implicated circular RNAs (circRNAs) in the progression of multiple malignant tumors. Nonetheless, the functions of circRNAs in NSCLC remain unclear. To study new targets for the treatment of NSCLC, circRNA expression profiling was performed on NSCLC tissues and para-carcinoma nonmalignant tissues. RNA was isolated and used for circRNA sequencing. Biological studies were performed *in vitro* and *in vivo* to determine the functions of circRNAs in NSCLC, including their functions in cell proliferation and migration. How circRNAs function in NSCLC was explored to clarify the underlying regulatory mechanisms. We found that circUCP2 was upregulated in NSCLC tissues compared with neighboring nonmalignant tissues. circUCP2 promoted the proliferation and metastasis of NSCLC cells. circUCP2 promoted NSCLC progression by sponging miR-149 and upregulating UCP2. The circUCP2/miR-149/UCP2 axis accelerates the progression of NSCLC, and circUCP2 may therefore be a novel diagnostic biomarker for the progression of NSCLC.

## Introduction

Lung cancer is a highly lethal cancer worldwide, and lung cancer accounts for almost 25% of all cancer-related deaths [1–3]. In particular, non-small cell lung cancer (NSCLC) accounts for 85% of all lung cancer cases [4]. In the last 20 years, substantial and promising progress has been made in NSCLC research. However, major challenges remain.

Increasing numbers of studies indicate that abnormal epigenetic modulation by noncoding RNAs might be involved in tumorigenesis [5]. Circular RNAs (circRNAs) are RNA molecules with covalently closed single chains that are generated by the back-splicing of pre-mRNA molecules [6]. CircRNAs play key roles in cancer development and progression and may be promising biomarkers or treatment targets [7]. In cancer, circRNAs have been implicated in cell proliferation, migration, invasion and survival [8,9]. In NSCLC, circRNAs can act as tumor promoters or suppressors. For example, hsa_circRNA_101237 promotes NSCLC proliferation, migration and invasion by sponging miR-490-3p and increasing the expression of MAPK [10]. On the other hand, circNDUFB2 inhibits NSCLC growth and metastasis by modulating IGF2BP degradation and antitumor immune responses [11].

To investigate novel therapeutic targets in NSCLC, we performed circRNA expression profiling on NSCLC tissues and para-carcinoma nonmalignant tissues [12]. RNA was isolated and subje to circRNA sequencing.

We found that circUCP2 was upregulated in NSCLC tissues *vs*. neighboring nonmalignant tissues. Moreover, circUCP2 enhanced NSCLC cell proliferation and metastasis *in vitro* and *in vivo*. circUCP2 sponged miR-149 to upregulate UCP2 and promote NSCLC progression. Therefore, circUCP2 may be a diagnostic marker as well as a therapeutic target for NSCLC.

## Materials and Methods

### Clinical sample collection and circRNA expression profile

Fresh NSCLC tissue samples and corresponding para-carcinoma nonmalignant tissue samples were collected at Sun Yat-sen University Cancer Center and immediately stored in liquid nitrogen. Total RNA was isolated and subjected to circRNA microarray analysis. The research protocols involving human samples and animals were approved by the Ethics Committee of Sun Yat-sen University Cancer Center and were performed according to the Declaration of Helsinki. All patients were informed and signed consent forms.

### Cell culture and transfection

Normal lung cells (Beas2b cells) and NSCLC cells (PC9, H1299, H1975 and A549 cells) were purchased from ATCC (USA). Cell authenticity was confirmed by DNA fingerprinting. Assays were routinely performed to detect mycoplasma infection.

The Lipofectamine 3000 system (Invitrogen, USA) was used to perform transfection. circUCP2 siRNAs, miR-149 mimics, and inhibitors were supplied by Ruibo (China). The siRNA sequences were as follows: si-NC, UUCUCCGAACGUGUCACGUTT; si-circUCP2 #1, ACCTTGGGGACCTCTCCCAAT; si-circUCP2 #2, AGCTCAACCTTGGGGACCTCT; si-circUCP2 #3, AACCTTGGGGACCTCTCCCAA.

### qRT‒PCR analysis

Total RNA isolation was completed using TRIzol (Sigma, USA). Nuclear and cytoplasmic RNA isolation was completed by Beyotime (China). Takara PrimeScript^TM^ RT reagent Kit and TB Green Premix Ex Taq^TM^ (Japan) were used for RT‒qPCR assays. circRNA levels were quantified via a method that was similar to the method for quantifying of mRNA levels. RNase R removal eliminated the effects of linear RNA. The 2^−ΔΔCt^ method was applied to calculate the expression levels of circRNAs and mRNAs. U6 and GAPDH were used as the controls for circRNA and mRNA expression, respectively. The following oligonucleotide primers were supplied by Ruibo (China): GAPDH, Forward (5′-3′), TGTTCGTCATGGGTGTGAAC, Reverse (5′-3′), ATGGCATGGACTGTGGTCAT; 18s, Forward (5′-3′), TTAATTCCGATAACGAACGAGA, Reverse (5′-3′), CGCTGAGCCAGTCAGTGTAG; UCP2, Forward (5′-3′), GGAGGTGGTCGGAGATACCAA, Reverse (5′-3′), ACAATGGCATTACGAGCAACAT; circUCP2, Forward (5′-3′), TCAGCCAGAATCTTCGTCCT, Reverse (5′-3′), CATAGGTCACCAGCTCAGCA.

### RNase R digestion

RNA (2 μg) was isolated from PC9 and H1975 cells and incubated with or without 3 U/μg RNase R at 37°C for 20 min. Then, the RNAs were purified with an RNeasy MinElute Cleanup Kit (Qiagen, USA) and subjected to qRT‒PCR.

### Actinomycin D assay

To block transcription, PC9 and H1975 cells were treated with 2 μg/mL actinomycin D (Sigma, USA) for 8, 16, and 24 h. Then, the cells were harvested, and the stability of circUCP2 and UCP2 mRNA was measured by qRT‒PCR.

### Cell counting kit-8 assay (CCK-8)

Ninety-six-well plates were seeded with 10^3^ cells after transfection, and then, the cells were incubated under proper conditions for 48 h. Then, 10 μL CCK-8 solution (Dojindo, Japan) was added to the culture medium and incubated for 2 h. The OD values were measured at 450 nM on a Bio-Tek EPOCH2 microtiter plate reader (USA), and the data were recorded.

### Colony formation assay

Six-well plates were seeded with 10^3^ cells after transfection. After incubation for 14 days at 37°C, when the clones were visible to the naked eye, the clones were fixed with paraformaldehyde, stained with crystal violet, and counted under a microscope.

### Transwell invasion assay

For the invasion assay, 10^4^ cells were seeded into upper chambers that were precoated with Matrigel (BD Bioscience, USA) and incubated in serum-free medium. Medium supplemented with 10% fetal bovine serum was added to the lower chambers. Twenty-four hours later, the cells that had invaded were fixed in cold methanol and stained with 1% crystal violet, and then, the number of stained cells were counted under a microscope.

### Mouse xenograft model

H1975 cells *(*2 × 10^6^) were subcutaneously administered into male nude mice (n = 5, five weeks old). Then, si-NC and si-circUCP2 were injected into the tumors every four days (40 μL). Twenty-eight days later, the tumors were excised, weighed, and subsequently subjected to pathological studies. A total of 10^5^ H1975 cells were injected into each mouse through the tail vein (n = 5, five weeks old). After 8 weeks, the lungs were excised for subsequent pathological studies. The number of metastatic sites in the lungs were counted under a microscope.

### Luciferase reporter assay

A 96-well plate was seeded with 3 × 10^4^ cells per well. Luciferase reporter plasmids carrying wild-type or mutant sequences of circUCP2 or UCP2 3′-UTR, miR-149 mimics, miR-149 inhibitors and their controls were purchased and cotransfected into cells. The activities of Renilla and firefly luciferase were measured 48 h later (Promega, USA).

### RNA immunoprecipitation (RIP)

MS2bs-circUCP2, MS2bs-circUCP2-mt and MS2bs-Rluc were transfected into NSCLC cells. Forty-eight hours later, the Magna RIP RNA-Binding Protein Immunoprecipitation Kit (Millipore, USA) was used for the RIP test. The beads were purified using the RNA-quick Purification Kit (Yishan, China) to obtain the eluted RNAs. qRT‒PCR assays were used to measure the levels of circUCP2, UCP2 and miR-149.

### Statistical analyses

Statistical analyses were performed with SPSS 20.0 software. *t*-tests were used to compare the differences between groups. All the data are presented as the mean ± SD. Statistical significance was set at *p* < 0.05.

## Results

### circUCP2 is expressed at high levels in NSCLC

**FIGURE 1:**
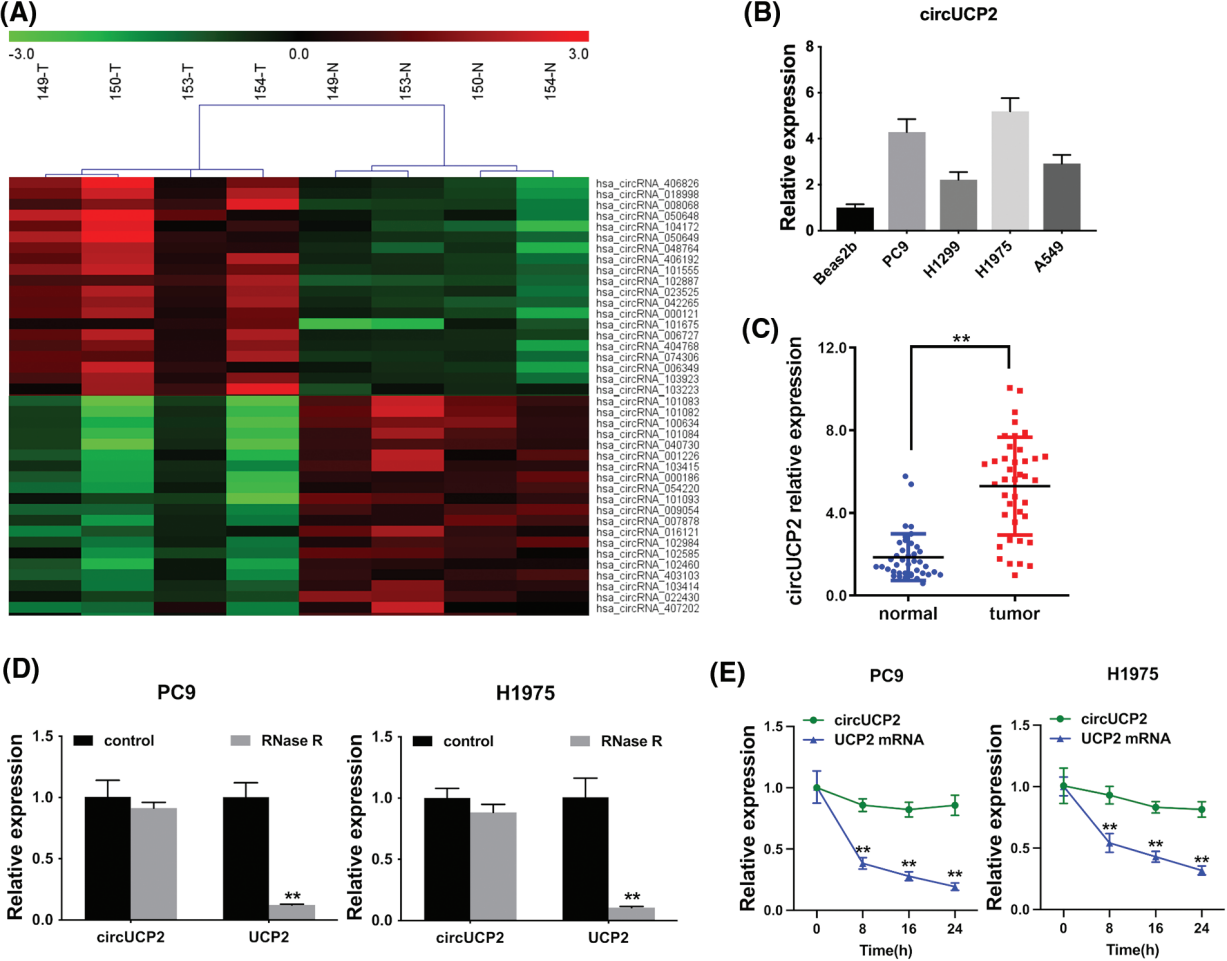
circUCP2 is upregulated in NSCLC. (A) The top 20 downregulated and upregulated circRNAs in NSCLC tissues (n = 4) compared with nonmalignant neighboring tissues (n = 4): red indicates upregulated circRNAs; green indicates downregulated circRNAs. (B) circUCP2 expression in NSCLC cells. (C) circUCP2 expression in 40 pairs of NSCLC tissues and neighboring nonmalignant tissues. (D) qRT‒PCR analysis of circUCP2 and UCP2 mRNA expression in PC9 and H1975 cells after treatment with RNase R. (E) qRT‒PCR analysis of circUCP2 and UCP2 mRNA expression in PC9 and H1975 cells after treatment with actinomycin D. **p < 0.01.

A high-throughput circRNA microarray from four matched NSCLC tissue samples and neighboring nonmalignant tissue samples was used to analyze the expression of circRNAs. The 20 most significantly upregulated and 20 most downregulated circRNAs are shown in Fig. 1A. We established that the hsa_circ_0023525 content was higher in NSCLC tissues than in neighboring nonmalignant tissues. According to the human reference genome (GRCh37/hg19), hsa_circ_0023525 is located at chr11:73685715-73687787 and originates from the uncoupling protein 2 (UCP2) gene. Thus, we named it “circUCP2”. Further experiments showed that circUCP2 is expressed at high levels in NSCLC cells (Fig. 1B). Then, we assessed circUCP2 expression in 40 pairs of NSCLC tissues and their controls. The data showed that circUCP2 was overexpressed in NSCLC tissues (Fig. 1C). To further confirm that circUCP2 is a circRNA, its resistance to digestion by the exonuclease RNase R was determined. As shown in Fig. 1D, the expression levels of the circRNA isoform transcript circUCP2 were not affected by RNase R, while the expression levels of the linear transcript UCP2 were significantly decreased. Moreover, actinomycin D assays further confirmed that the half-life of circUCP2 was more than 24 h, showing that it was far more stable than the linear transcript UCP2 in NSCLC cells (Fig. 1E).

### Silencing of circUCP2 represses cell proliferation along with invasion

**FIGURE 2:**
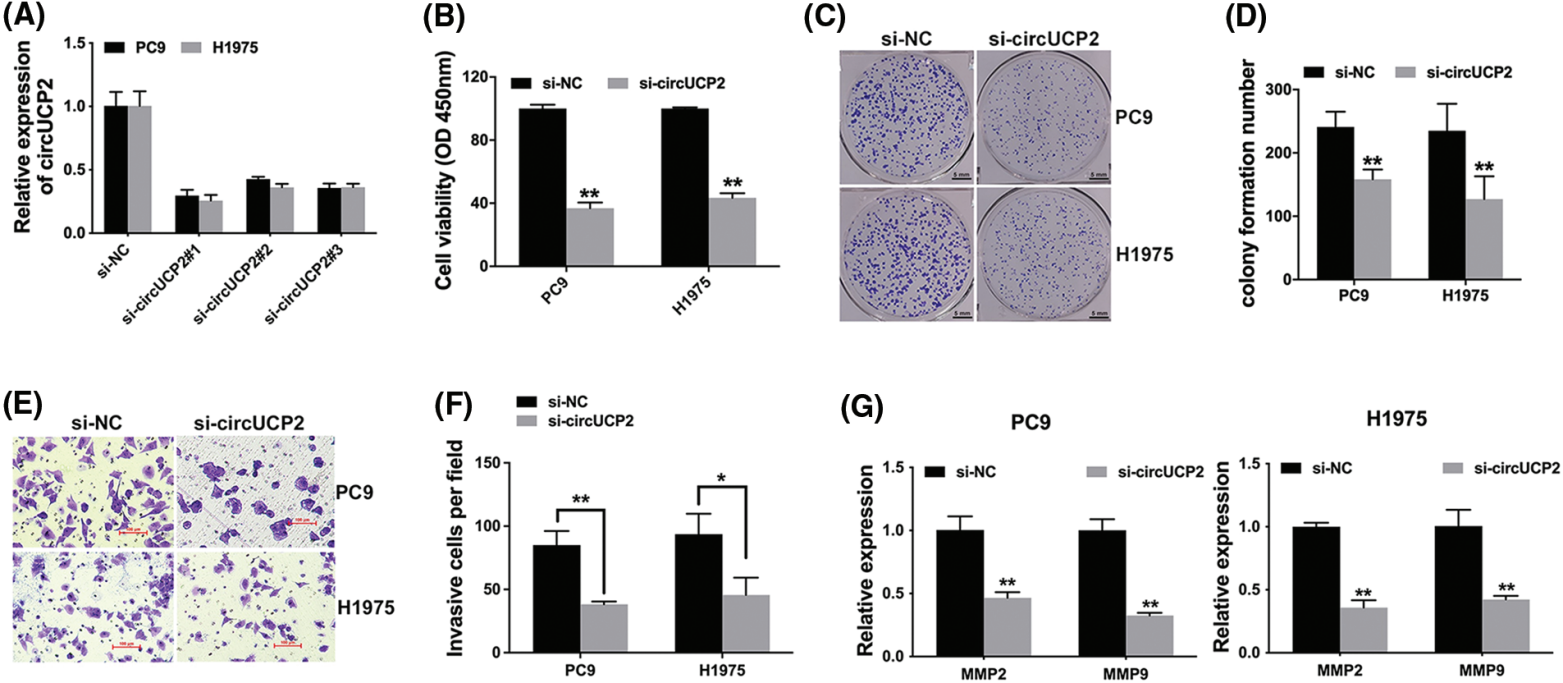
Silencing of circUCP2 represses cell proliferation along with invasion. (A) siRNAs were used to knockdown circUCP2 expression. (B) CCK-8 assays were used to evaluate the proliferation of NSCLC cells. (C) Colony formation assays were used to assess colony formation. (D) Statistical analysis of number of colonies formed. (E) The invasive ability of cells was tested. (F) Statistical analysis of invasive cell numbers. (G) qRT‒PCR measurement of the expression levels of MMP2 and MMP9 in NSCLC cells. *p < 0.05, **p < 0.01.

We used siRNAs to knockdown the expression of circUCP2, and si-circUCP2#1 was selected for subsequent experiments (Fig. 2A). We carried out a CCK-8 assay and established that circUCP2 silencing suppressed cell proliferation (Fig. 2B). Additionally, knockdown of circUCP2 inhibited NSCLC cell colony formation (Fig. 2C and 2D). Moreover, the invasive ability of NSCLC cells was repressed when circUCP2 was knocked down (Figs. 2E and 2F). Additionally, we measured the expression of MMP2 and MMP9, which are indicators of cancer metastasis. Knockdown of circUCP2 suppressed the expression levels of both MMP2 and MMP9 in NSCLC cells (Fig. 2G).

### Silencing of circUCP2 represses cell proliferation along with metastasis

**FIGURE 3:**
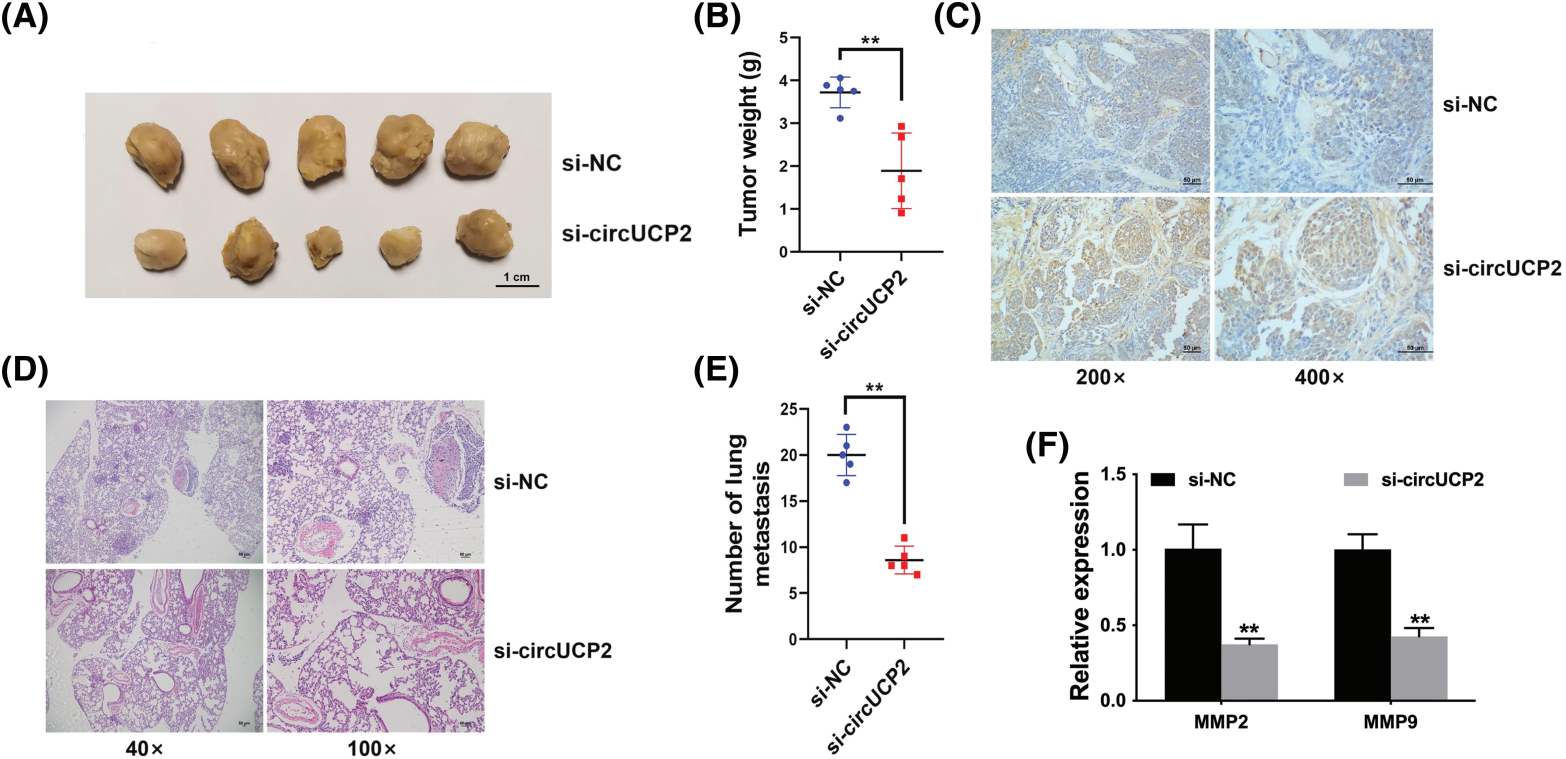
Silencing of circUCP2 represses cell proliferation along with metastasis. (A) Mouse xenograft models were used to evaluate the roles of circUCP2. (B) Statistical analysis of tumor weights. (C) IHC staining for Ki67. (D) HE-staining of metastatic sites. (E) Statistical analysis of metastasis numbers. (F) qRT‒PCR measurement of the expression levels of MMP2 and MMP9 in xenograft tumor tissues. **p < 0.01.

Silencing of circUCP2 repressed growth ability in mouse xenograft model experiments (Figs. 3A and 3B). Further immunohistochemistry (IHC) staining for Ki67 showed that Ki67 expression was decreased in circUCP2-knockdown tumor tissues, indicating that circUCP2 inhibition reduced cell proliferation in xenograft tumors (Fig. 3C). Moreover, knockdown of circUCP2 repressed metastasis in mice (Figs. 3D and 3E). In addition, we measured the expression of MMP2 and MMP9 and found that the expression levels of both MMP2 and MMP9 were decreased in circUCP2-knockdown tumor tissues (Fig. 3F).

### circUCP2 acts as a miR-149 sponge in NSCLC

**FIGURE 4:**
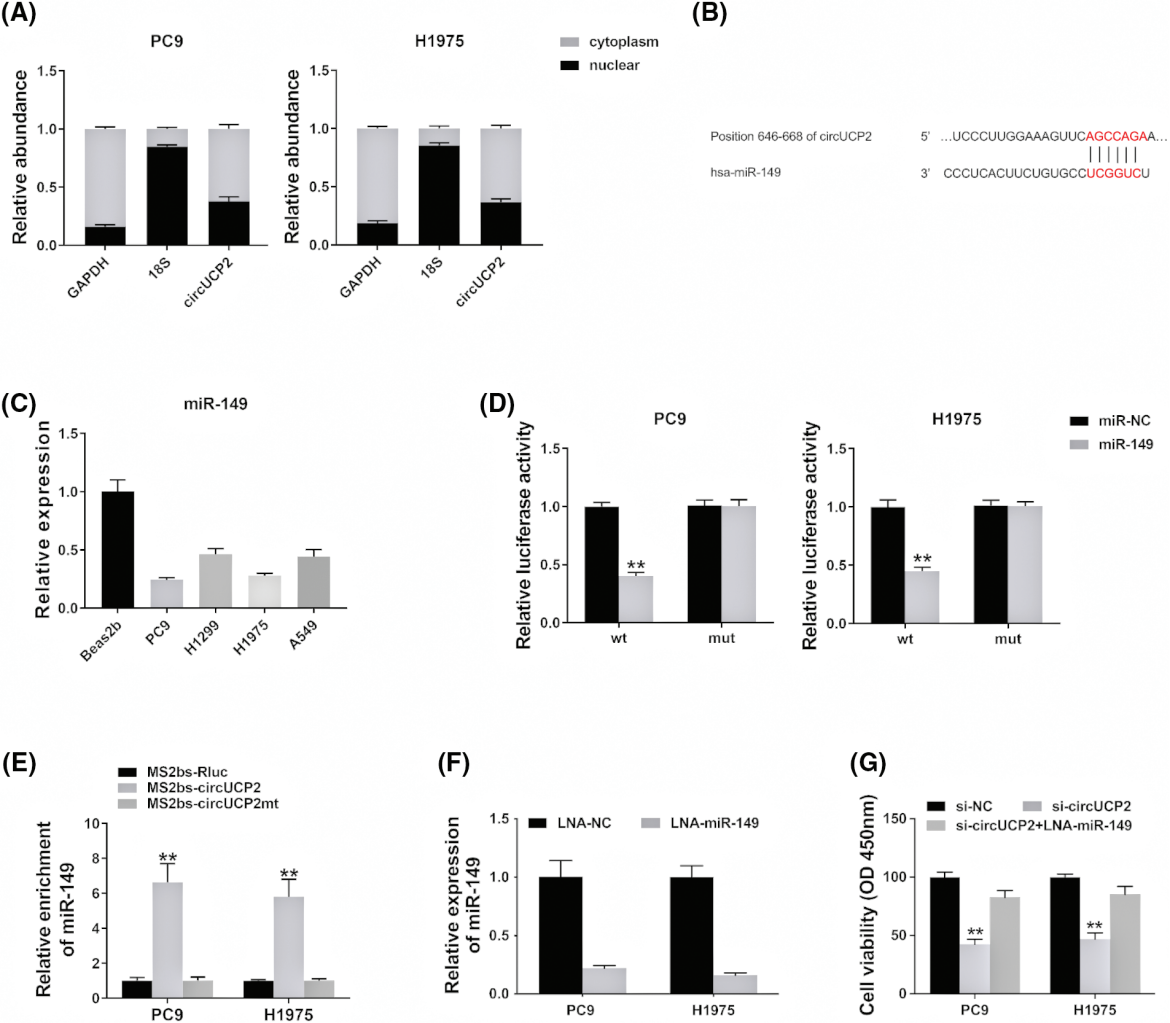
circUCP2 acts as a miR-149 sponge in NSCLC. (A) circUCP2, UCP2, 18S and GAPDH expression levels in the cytoplasm and nucleus. (B) Potential binding sites between circUCP2 and miR-149. (C) miR-149 expression levels in NSCLC cells. (D) Luciferase assay was performed. (E) MS2-based RIP assay was performed in different groups. (F) LNA was used to knockdown miR-149 expression. (G) CCK-8 assay was used to evaluate the proliferation ability of NSCLC cells. **p < 0.01.

Recent studies have shown that circRNAs repress miRNA expression by sequestering miRNAs [13]. Some circRNAs in the cytoplasm act as competing endogenous RNAs (ceRNAs) and sponge miRNAs to prevent them from binding to and repressing their target mRNAs [14]. Thus, we evaluated the cellular location of circUCP2 in NSCLC cells. The data showed that circUCP2 was primarily localized in the cytoplasm (Fig. 4A), which could allow it to function as an miRNA sponge. Thus, we searched CircInteractome to find possible circRNA/miRNA interactions, and miR-149 was predicted. The docking sites of miR-149 in the circUCP2 sequence are shown in Fig. 4B. Next, we explored the level of miR-149 in PC9, H1299, H1975 and A549 NSCLC cells, and the miR-149 content was found to be decreased (Fig. 4C). The luciferase enzyme reporter assay revealed a reduced luciferase intensity in cells cotransfected with plasmid containing wild-type sequences, as well as miR-149 mimics (Fig. 4D). To further investigate the direct docking between circUCP2 and miR-149, we performed an RIP assay. The results revealed that miR-149 was most abundant in the MS2bs-circUCP2 group (Fig. 4E), showing that circUCP2 could directly cross talk with miR-149 and sponge miR-149. Finally, we inhibited the expression of miR-149 with LNA in NSCLC cells to confirm the functions of miR-149 (Fig. 4F). The CCK-8 assay revealed that circUCP2 silencing suppressed cell proliferation, while inhibition of miR-149 reversed these effects of circUCP2 (Fig. 4G), indicating that circUCP2 functioned partly by regulating miR-149 in NSCLC.

### circUCP2 functions as a ceRNA to modulate UCP2

**FIGURE 5:**
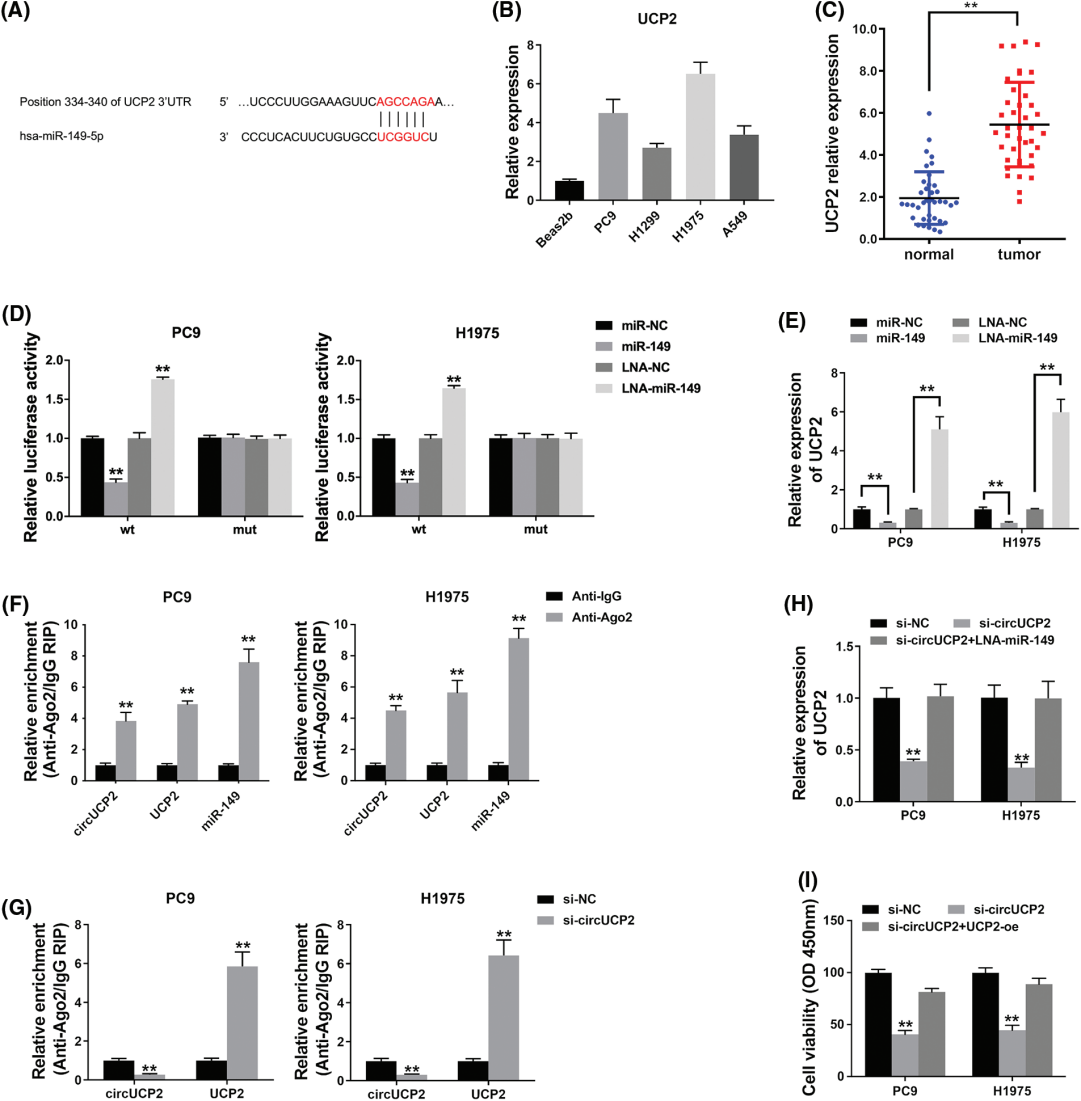
circUCP2 functions as a ceRNA to modulate UCP2. (A) Docking sites within the UCP2 3′UTR and miR-149 were predicted. (B) qRT‒PCR was used to measure UCP2 expression in NSCLC cells. (C) UCP2 expression levels in 40 NSCLC tissues and their control samples. (D) Luciferase assay was used to detect binding relationships. (E) UCP2 expression levels measured after the transfection of miR-149 and its suppressor. (F) The enrichment of circUCP2, UCP2 and miR-149 by Ago2. (G) The enrichment of UCP2 after circUCP2 silencing. (H) UCP2 expression levels in cells were measured. (I) CCK-8 assay was used to evaluate the proliferation ability of NSCLC cells. **p < 0.01.

To investigate whether circUCP2 sponges miR-149 to allow the expression of downstream targets, a possible binding site of miR-149 was searched using TargetScan. The docking site of miR-149 in the UCP2 3′UTR was predicted (Fig. 5A). Therefore, we assessed UCP2 expression in NSCLC cells and tissues and found that it was overexpressed (Figs. 5B and 5C). A luciferase enzyme reporter assay revealed a reduced luciferase intensity in cells that were cotransfected with wild-type luciferase reporter and miR-149 mimics (Fig. 5D). Furthermore, the luciferase intensity was elevated in cells cotransfected with the wild-type luciferase reporter and miR-149 inhibitor (Fig. 5D). Next, we examined UCP2 expression in NSCLC cells and found that miR-149 could inhibit UCP2 expression and that the miR-149 suppressor promoted UCP2 expression levels (Fig. 5E). The results verified that miR-149 regulated UCP2 expression levels.

Ago2 was predominantly enriched by circUCP2, UCP2 and miR-149 in the RIP assay (Fig. 5F), which indicated that circUCP2 as well as UCP2 can be mobilized to Ago2-linked RISC followed by binding to miR-149. In addition, circUCP2 silencing decreased circUCP2 binding to Ago2 while promoting UCP2 binding (Fig. 5G), which illustrated that circUCP2 bound to miRNAs by competing with UCP2. Silencing of circUCP2 reduced UCP2 expression, but miR-149 inhibitor cotransfection reversed the effect (Fig. 5H); this indicated that circUCP2 sponged miR-149 to regulate UCP2 expression in NSCLC. sssMoreover, the CCK-8 assay revealed that circUCP2 silencing suppressed cell proliferation, while overexpression of UCP2 reversed these effects of circUCP2 (Fig. 5I), indicating that circUCP2 functioned partly by regulating UCP2 in NSCLC.

## Discussion

NSCLC is the most common deadly disease, and better treatments are urgently needed. CircRNAs are closely associated with NSCLC progression [15]. CircRNA F-circEA-2a is reported to enhance NSCLC cell migration along with invasion [16]. The circRNAs F-circSR1 and F-circSR2 enhance NSCLC cell migration [17]. Here, we found that in contrast to neighboring nonmalignant tissues, circUCP2 is upregulated in NSCLC tissues (Fig. 1). Further assays showed that silencing circUCP2 inhibited NSCLC cell growth along with metastasis, indicating its indispensable role in NSCLC (Figs. 2 and 3).

Recent reports have shown that miRNA activity is affected by transcripts of miRNA sponges, so-called ceRNAs [18]. CircRNAs function as oncogenes or anti-oncogenes by binding to their target miRNAs. In NSCLC, circPTK2 acts as a ceRNA to repress cell infiltration by regulating miR-429/miR-200b-3p [19]. In addition, circFGFR1 sponges miR-381-3p to elevate CXCR4 expression to promote NSCLC progression and therapy resistance [20]. Here, we showed that circUCP2 had the potential to sponge miR-149 by directly binding to it (Fig. 4). In addition, circUCP2 functioned partly by regulating miR-149 in NSCLC.

miR-149 has been widely reported to function as a tumor repressor. miR-149 expression levels are dramatically repressed in cancer-associated fibroblasts (CAFs) in gastric cancer. miR-149 inversely modulates CAFs, as well as their influence on the development of GC, by targeting IL-6 [21]. In hepatocellular carcinoma, miR-149 serves as a tumor inhibitory miRNA and inhibits tumorigenesis by regulating the AKT/mTOR cascade [22]. In breast cancer, miR-149 acts as a metastasis-repressing microRNA by limiting the colony-stimulating factor-1 (CSF1)-dependent mobilization of macrophages as well as M2 macrophage polarization [23].

miR-149 has been reported to be downstream of the ceRNA mechanism. In GC cells, circNRIP1 acts as a tumor promotor and functions as a sponge of miR-149 to influence AKT1 expression. miR-149 hinders the function of circNRIP1 during the progression of malignancy [24]. CircCTNNA1 can absorb miR-149 and antagonize its repressive influence on its subsequent target Forkhead Box M1 (FOXM1) to enhance cell proliferation and infiltration in colon cancer [25]. Moreover, circ5615 can sponge miR-149 and activate the Wnt/β-catenin cascade [26].

It has been documented that miR-149 participates in the pathogenesis of NSCLC [27]. LncRNA MIAT is reported to sponge miR-149 and increase FOXM1 expression in NSCLC [28]. LncRNA PCAT-1 upregulates LRIG2 expression by competitively sponging miR-149 and promotes NSCLC development [29]. Moreover, lncRNA HNF1A-AS1 sponges miR-149 and upregulates Cdk6 to promote NSCLC tumorigenic ability [30]. Here, we showed that circUCP2 functioned as a ceRNA by sponging miR-149 and upregulating UCP2 expression in NSCLC (Fig. 5) and that circUCP2 functioned partly by regulating UCP2 in NSCLC.

UCP2 represses reactive oxygen species (ROS) production by mitochondria, and it is involved in diverse physiological and pathological processes, e.g., the differentiation of stem cells along with cancer [31]. UCP2 is often highly expressed in cancer cells that are resistant to drugs to inhibit ROS accumulation and apoptosis [32]. UCP2-overexpressing tumor xenografts continue to grow after chemotherapy, which links UCP2 to chemoresistance [33]. In pancreatic adenocarcinoma, UCP2 inhibition strongly increases ROS production and induces autophagy, which inhibits cell growth and triggers ROS-dependent cell death [34]. In breast cancer, there is a significant relationship between UCP2 and tumor grade. UCP2 silencing strongly induces cell apoptosis and suppresses cell survival and proliferation [35]. Moreover, miR-214 increases breast cancer cell sensitivity by targeting UCP2 and inhibiting cell autophagy [36]. Targeting UCP2 could be a potential strategy for cancer treatment. Nevertheless, its biochemical and physiological roles in NSCLC remain unclear.

Here, we established that UCP2 was overexpressed in NSCLC cells and tissues. In addition, it was regulated by miR-149. Further mechanistic study showed that circUCP2 functioned as a ceRNA by sponging miR-149 and upregulating UCP2 expression, leading to NSCLC tumor progression.

## Data Availability

All data used and analyzed in this study are available from the corresponding author on reasonable request. Email could be sent to the address below to obtain the shared data: liuq1@sysucc.org.cn; yanghan@sysucc.org.cn.
